# A Study of Sponge Symbionts from Different Light Habitats

**DOI:** 10.1007/s00248-023-02267-x

**Published:** 2023-08-19

**Authors:** D. F. R. Cleary, N. J. de Voogd, T. M. Stuij, T. Swierts, V. Oliveira, A. R. M. Polónia, A. Louvado, N. C. M. Gomes, F. J. R. C. Coelho

**Affiliations:** 1 CESAM & Department of Biology, University of Aveiro, Campus de Santiago, 3810-193 Aveiro, Portugal; 2 Naturalis Biodiversity Center, Leiden, The Netherlands; 3 Institute of Environmental Sciences (CML), Leiden University, Leiden, The Netherlands

**Keywords:** 16S, Caribbean, Composition, Martinique, Porifera

## Abstract

The amount of available light plays a key role in the growth and development of microbial communities. In the present study, we tested to what extent sponge-associated prokaryotic communities differed between specimens of the sponge species *Cinachyrella kuekenthali* and *Xestospongia muta* collected in dimly lit (caves and at greater depths) versus illuminated (shallow water) habitats. In addition to this, we also collected samples of water, sediment, and another species of *Cinachyrella*, *C. alloclada*. Overall, the biotope (sponge host species, sediment, and seawater) proved the major driver of variation in prokaryotic community composition. The light habitat, however, also proved a predictor of compositional variation in prokaryotic communities of both *C. kuekenthali* and *X. muta*. We used an exploratory technique based on machine learning to identify features (classes, orders, and OTUs), which distinguished *X. muta* specimens sampled in dimly lit versus illuminated habitat. We found that the classes Alphaproteobacteria and Rhodothermia and orders Puniceispirillales, Rhodospirillales, Rhodobacterales, and Thalassobaculales were associated with specimens from illuminated, i.e., shallow water habitat, while the classes Dehalococcoidia, Spirochaetia, Entotheonellia, Nitrospiria, Schekmanbacteria, and Poribacteria, and orders Sneathiellales and Actinomarinales were associated with specimens sampled from dimly lit habitat. There was, however, considerable variation within the different light habitats highlighting the importance of other factors in structuring sponge-associated bacterial communities.

**Supplementary Information:**

The online version contains supplementary material available at 10.1007/s00248-023-02267-x.

## Introduction

The widespread application of DNA sequencing technologies has revealed a diversity of microorganisms far greater than previously thought. This has led to a fundamental shift in our understanding of animal-microbe associations with multicellular organisms no longer being considered fully autonomous entities but holobionts [1]. Sponges, one of the oldest multicellular animal lineages, have been extensively studied as model holobionts in marine environments. In addition to being model organisms of the early evolution of host-microbe symbioses, sponges also play important structural roles in coral reefs where they provide living environments for several other marine species and bridge the benthic and pelagic zones with their filtering activities [2]. They are also prolific sources of bioactive natural products, many of which are produced by their microbial symbionts [3, 4].

Several spatial and environmental processes, e.g., temperature, salinity, chlorophyll a concentrations, and the degree of light illumination, affect the composition and functioning of microbial communities including those associated with sponges [5–20]. Light is of particular importance to marine microbial communities. In the marine environment, light levels decrease from brightly illuminated surface waters to more dimly lit conditions at greater depths and, in caves, from the cave entrance to the dark recesses of the cave interior. This reduction in light levels has a direct impact on several environmental processes in aquatic environments [21–25].

Here, we compared prokaryotic communities of *X. muta* and one species of *Cinachyrella* (*C. kuekenthali*) sampled in dimly lit (caves and at greater depths) and illuminated (shallow water) habitats surrounding the island of Martinique. Both of these species span a gradient from shallow to mesophotic waters thereby providing models to study the effects of light attenuation on sponge-associated prokaryotic communities [13, 26, 27]. In addition to this, we also sampled sediment, water, and specimens of another shallow water *Cinachyrella* species, *C. alloclada*. The giant barrel sponge *X. muta* has been previously assigned high microbial abundance (HMA) status [28–30]. It is one of the most abundant species in Caribbean reefs [31]. Sponges of the genus *Cinachyrella* are common in the coastal waters of Florida and the Caribbean. *Cinachyrella alloclada* and *Cinachyrella kuekenthali* are sympatric species in Martinique and also elsewhere in the wider Caribbean basin [32]. However, unlike the shallow water species C. *alloclada*, *C. kuekenthali* has been observed along a depth range from 0.2 to 100 m [33]. Species of the genus *Cinachyrella* are often covered by sediment and algae and can look very similar; they can, thus, be challenging to differentiate in the field without careful skeletal observation. Members of this genus appear to have marked differences in their associated microbial communities [34].

The main underlying hypothesis of the present study is that sponges in different light habitats host distinct prokaryotic communities. In addition to this, we aimed to assess if different sponge species, sediment, and seawater also housed distinct prokaryotic communities, and to identify taxa associated with different light habitats in the sponge species *X. muta*.

## Materials and Methods

### Sample Collection

**Fig. 1:**
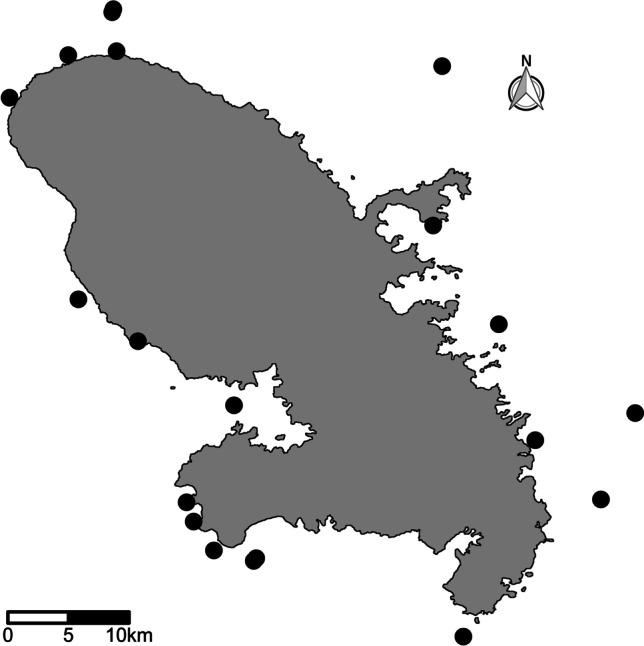
Map of Martinque showing the locations of the sampling sites

In the present study, we collected 34 samples of three sponge species, four sediment, and three water samples between 3 and 90 m depth from various sites around the island of Martinique (Fig. 1) from September 5th till October 10th, 2016. The shallow water and cave samples were collected between 3 and 31 m depth using SCUBA diving or snorkeling and the samples from deeper water were collected by dredging. The deeper samples consisted of three *C. kuekenthali* and nine *X. muta* specimens. These were all sampled from depths greater than 60 m. The dredge consisted of a triangular metal frame of 30 cm with a 1 mm mesh net, which was connected to a rope with a length of 200 m and pulled by a light boat. Each dredge lasted between 5 and 20 min depending on the type of background. Sponges were handpicked from the dredge, photographed and preserved in 96% ethanol. Additional data on all samples collected including the geographical coordinates of the sample sites and depths are provided in Supplementary data 1.

**Fig. 2:**
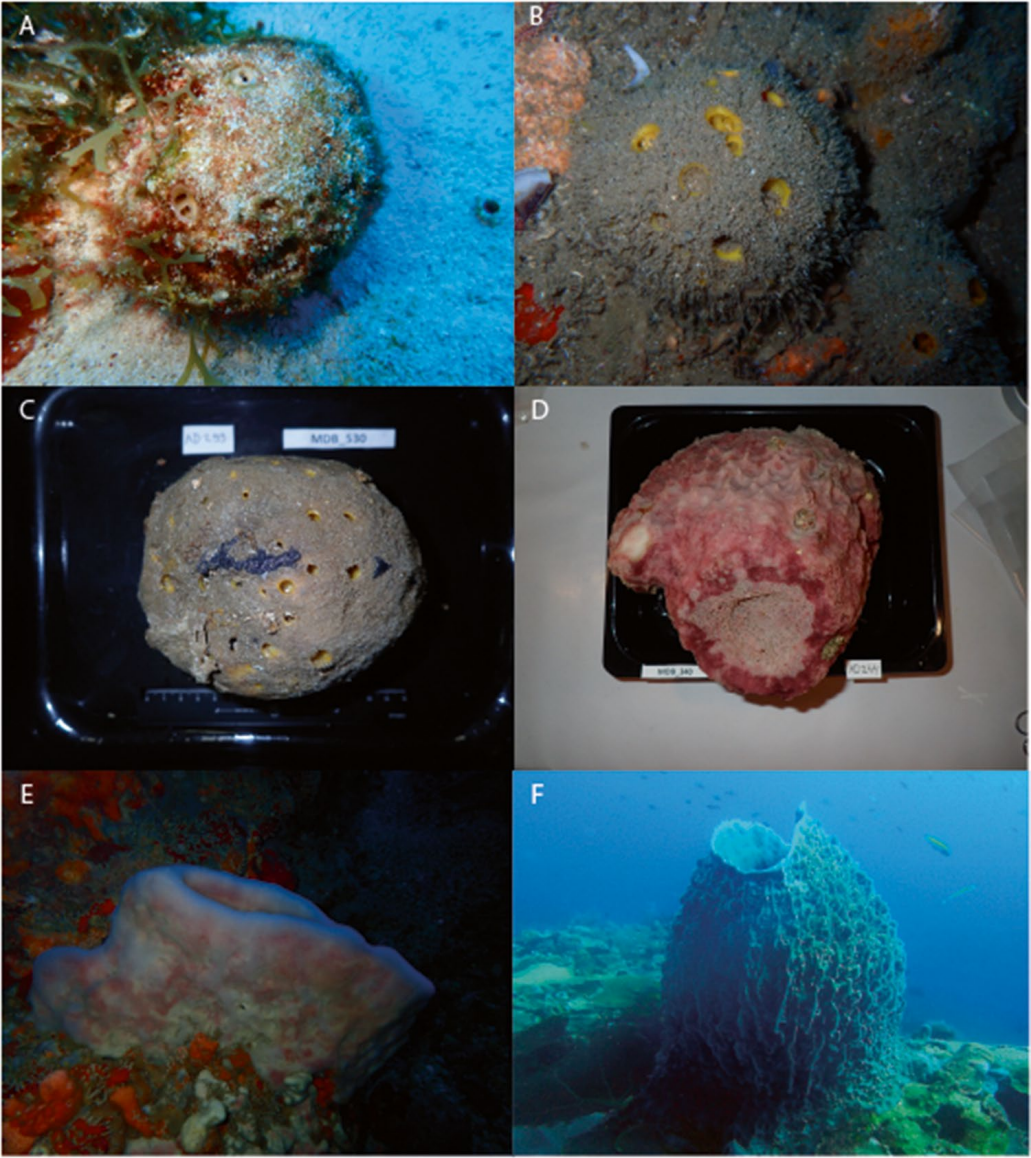
Selected photographs of the focal sponge species., **A** *Cinachyrella alloclada*, **B** *Cinachyrella kuekenthali* sampled from shallow water, **C** deep water, *Xestospongia muta* sampled from **D** deep water, **E** a cave, and **F** shallow water. The samples in **C** and **D** were collected using dredging and the pictures were taken in the remote lab

Twelve samples of *Xestospongia muta* (Schmidt, 1870) were collected in shallow waters between 7 and 31 m, nine samples were collected in deeper waters, > 60 m, and three were collected from shallow caves between 14 and 21 m depth. In the present study, we pooled the cave specimens and specimens sampled at greater depths in order to distinguish dimly lit versus illuminated (shallow water) habitats. Specimens from caves and deeper water are, however, distinguished in the figures. *Xestospongia muta* is found along a shallow to mesophotic depth gradient [13]. Olson and Gao [14] initially identified *X. muta* as hosting a diverse microbial community including abundant Chloroflexi and cyanobacterial components. Four individuals of *Cinachyrella alloclada* (Uliczka, 1929) were collected between 3 and 22 m depth. Three individuals of *Cinachyrella kuekenthali* (Uliczka, 1929) were collected in shallow waters between 3 and 22 m and three in deeper waters (> 60 m). Photographs of selected sponge specimens are shown in Fig. 2.

Four sediment samples were collected from the top 5 cm surface layer in sites between 10 and 15 m depth using a Falcon tube. Three seawater samples were collected from sites between 2 and 5 m depth; 1 l was filtered through a Millepore white Isopore membrane filter (0.22 μm pore size) to obtain seawater prokaryotic communities. All samples were stored in ethanol (96%) and kept in a cooler until they were finally stored at −20 °C at the Naturalis Biodiversity Center. Voucher specimens of each sponge sample have been deposited at the sponge collection of the Naturalis Biodiversity Center, Leiden, The Netherlands (as RMNH POR).

#### DNA Extraction and Next-Generation Sequencing Analysis

PCR-ready genomic DNA was isolated from all samples using the FastDNA® SPIN soil Kit (MPbiomedicals) following the manufacturer’s instructions. Briefly, the whole membrane filters, for water samples, were cut into small pieces and transferred to Lysing Matrix E tubes containing a mixture of ceramic and silica particles; for the other samples, ± 500 mg of sponge tissue (including parts of the surface and interior) or sediment was added. The microbial cell lysis was performed in the FastPrep® Instrument (Q Biogene) for 80 s at 6.0 ms^−1^. The extracted DNA was eluted into DNase/Pyrogen-Free water to a final volume of 50 μl and stored at −20 °C until use. The 16S rRNA gene V3-V4 variable region PCR primers 341F 5’-CCTACGGGNGGCWGCAG-3’ and 785R 5’-GACTACHVGGGTATCTAATCC-3’ [35] with barcode on the forward primer were used in a 30 cycle PCR assay using the HotStarTaq Plus Master Mix Kit (Qiagen, USA) under the following conditions: 94 °C for 3 min, followed by 28 cycles of 94 °C for 30 s, 53 °C for 40 s, and 72 °C for 1 min, after which a final elongation step at 72 °C for 5 min was performed. After amplification, PCR products were checked on a 2% agarose gel to determine the success of amplification and the relative intensity of bands; the blank control did not yield any bands. PCR product was used to prepare the DNA library following the Illumina TruSeq DNA library preparation protocol. Next-generation, paired-end sequencing was performed at MrDNA (Molecular Research LP; http://www.mrdnalab.com/; last checked 18 November 2016) on an Illumina MiSeq device (Illumina Inc., San Diego, CA, USA) following the manufacturer’s guidelines. Sequences from each end were joined following Q25 quality trimming of the ends followed by reorienting any 3′–5′ reads back into 5′–3′ and removal of short reads (< 150 bp).

#### 16S rRNA Gene Sequencing Analysis

The 16S rRNA gene amplicon libraries were analyzed using QIIME2 (version 2019.7) [36]. Raw data was imported yielding a demultiplexed “qza” data file (artifact). The DADA2 plugin [37] in QIIME 2 was subsequently used to trim sequences (final length 400 nt). The DADA2 analysis yielded output archives containing an OTU (also known as amplicon sequence variant or “ASV”) table, denoising stats and a fasta file of representative sequences. The feature-classifier plugin with the extract-read method was then used with the i-sequences argument set to silva-138-99-seqs.qza. This was followed by the feature-classifier plugin with the fit-classifier-naive-bayes method and the i-reference-taxonomy method set to silva-138-99-tax.qza. Both silva-138 files can be obtained from https://docs.qiime2.org/2020.8/data-resources/?highlight=silva. The feature-classifier plugin was then used with the classify-sklearn method and the i-reads argument set to the representative sequences file generated by the DADA2 analysis to produce a table with taxonomic assignment for all OTUs. Finally, mitochondria, chloroplasts, and Eukaryota were filtered out using the qiime taxa plugin with the filter-table method. The OTU and taxonomy tables were later merged in R [38]. The DNA sequences generated in this study can be downloaded from NCBI BioProject Id: PRJNA715755.

#### Statistical Analyses

A table containing the OTU counts was imported into R using the read.csv function. Supplementary data 2 contains all operational taxonomic unit (OTU) counts per sample and taxonomic assignments of all OTUs. The 50 most abundant OTUs are presented in Supplementary data 3. The raw OTU counts matrix was used to compare diversity among groups and to test for differences in higher taxon abundances. Diversity indices were obtained using the rarefy and diversity functions from the vegan [39] package in R. Evenness was calculated by dividing Shannon’s H′ by the number of OTUs in each sample. We tested for significant differences in the relative abundance of the most abundant prokaryotic phyla and classes, OTU richness, evenness, Shannon’s H′, and Fisher’s alpha indices among groups (sediment, seawater, *C. alloclada*, and *C. kuekenthali* and *X. muta* from dimly lit and illuminated habitats thus forming seven groups/biotopes) with an analysis of deviance using the glm function in R. For the diversity indices, we set the family argument to “tweedie” using the tweedie function [40] in R with var.power=1.5 and link.power=0 (a compound Poisson–gamma distribution). For the relative abundances of higher taxa, we set the family argument to quasibinomial. Using the GLM model, we tested for significant variation among groups using the ANOVA function in R with the *F* test. We used the emmeans function in the emmeans library [41] in R to perform multiple comparisons of mean abundance among groups using estimated marginal means (EMM) with the false discovery rate (fdr) method in the adjust argument of the emmeans function and a *P* value of 0.05. Only groups with at least three samples were included.

Variation in prokaryotic composition among groups was assessed with principal coordinates analysis (PCO). We tested for significant differences among groups with an adonis analysis from the vegan package. For the PCO, a Bray-Curtis distance matrix was first obtained using the Phyloseq package [41] and the count data was rarefied using the rarefy_even_depth function with the sample.size argument set to the minimum sample size (13038 in the present study) and subsequently log_10_ transformed. A separate analysis was run including only specimens of *X. muta* in order to test for a significant effect of light habitat on prokaryotic composition.

We used an exploratory technique based on machine learning, Boruta, to identify features, which distinguished between *X. muta* specimens sampled in dimly lit (deeper and cave) versus illuminated (shallow water) habitat. Boruta, named after a slavic forest demon, is a random forest wrapper, which is used to evaluate feature importance [42]. The first step in the Boruta algorithm is the generation of a data frame consisting of randomly shuffled versions of all original features, which are termed shadow features. This data frame is then attached to the data frame of original features creating a new data frame with double the amount of features. A random forest machine learning algorithm is then applied to the new data frame consisting of all the original and shadow features in addition to the response variable. The importance of each original feature is then compared with a threshold based on the shadow features. This process is repeated a given number of times (permutations) producing a binomial distribution. Original features at the tails of the distribution are either rejected or accepted while those in the middle are considered tentative. Increasing the number of randomizations can help to resolve tentatively classified features. In the present study, we generated three data frames consisting of classes, orders, OTUs, and a response variable indicating whether a sample of *X. muta* was collected in dimly lit versus illuminated habitat. Prior to the Boruta analysis, highly correlated features (> 0.8) were removed from the data frames. The Boruta function from the Boruta package in R [43] was then used with the habitat (dimly lit versus illuminated) as response variable and orders, classes, or OTUs as features (predictive variables). The doTrace argument in the Boruta function was set to 2 and the maxRuns argument set to 1000; other arguments used default values. The number of significant features was identified and the six features (classes, orders, or OTUs) with the highest importance presented.

## Results

**Fig. 3:**
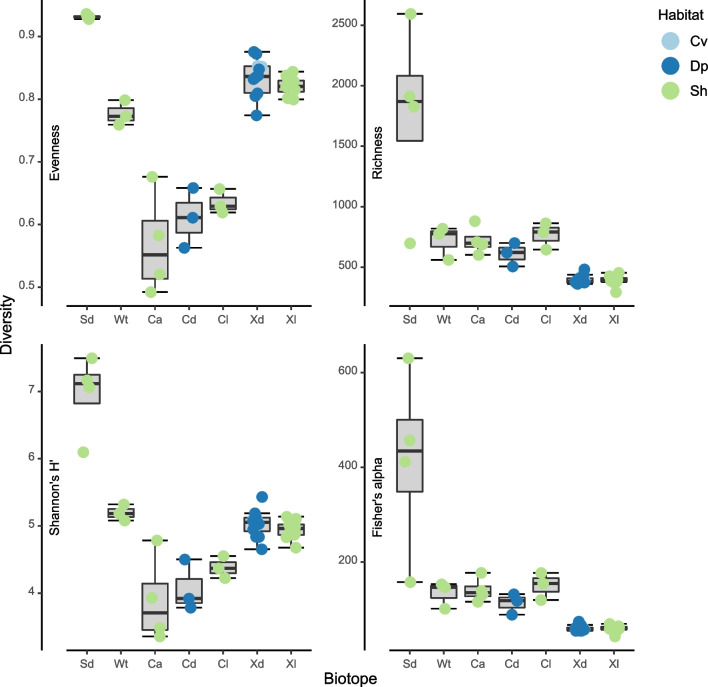
Boxplots showing values for selected diversity indices. Results of GLM analyses are shown after the respective index. Evenness: *F*_6,34_ = 56.48, *P* < 0.001, richness: *F*_6,34_ = 46.61, *P* < 0.001, Shannon: *F*_6,34_ = 35.78, *P* < 0.001, and Fisher: *F*_6,34_ = 65.65, *P* < 0.00. The x-axis labels refer to sediment (Sd), water (Wt), *Cinachyrella alloclada* (Ca), *Cinachyrella kuekenthali* from dimly lit (Cd) and illuminated (Cl) habitats, and *Xestospongia muta* from dimly lit (Xd) and illuminated (Xl) habitats. Colored symbols indicate specimens collected from caves (Cv), deep (Dp), and shallow water (Sh)

After quality control and removal of OTUs assigned to chloroplasts and mitochondria, the data set consisted of 3172098 sequences and 11977 OTUs. In terms of sequences, the most abundant phyla in the present study were Proteobacteria (1371433 sequences, 4556 OTUs), Chloroflexi (442626 sequences, 602 OTUs), Actinobacteriota (351143 sequences, 572 OTUs), and Myxococcota (143938 sequences, 314 OTUs). Rarefied richness and evenness were significantly higher in sediment than all other biotopes (Fig. 3 and Supplementary data 4). Evenness was also significantly higher in *X. muta* than *Cinachyrella* spp. Richness, in turn, was significantly lower in *X. muta* sampled from both dimly lit and illuminated habitat than in both *Cinachyrella* species. Shannon’s H′ and Fisher’s alpha diversity indices largely reflected results of evenness and rarefied richness, respectively (Supplementary data 4).

**Fig. 4:**
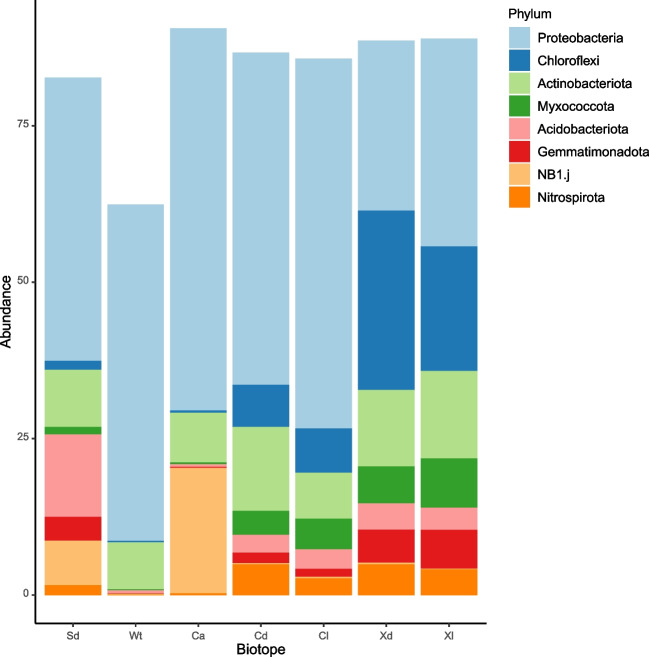
Stacked barplots of the mean relative abundances of the most abundant phyla. The x-axis labels refer to sediment (Sd), water (Wt), *Cinachyrella alloclada* (Ca), *Cinachyrella kuekenthali* from dimly lit (Cd) and illuminated (Cl) habitat, and *Xestospongia muta* from dimly lit (Xd) and illuminated (Xl) habitat

**Fig. 5:**
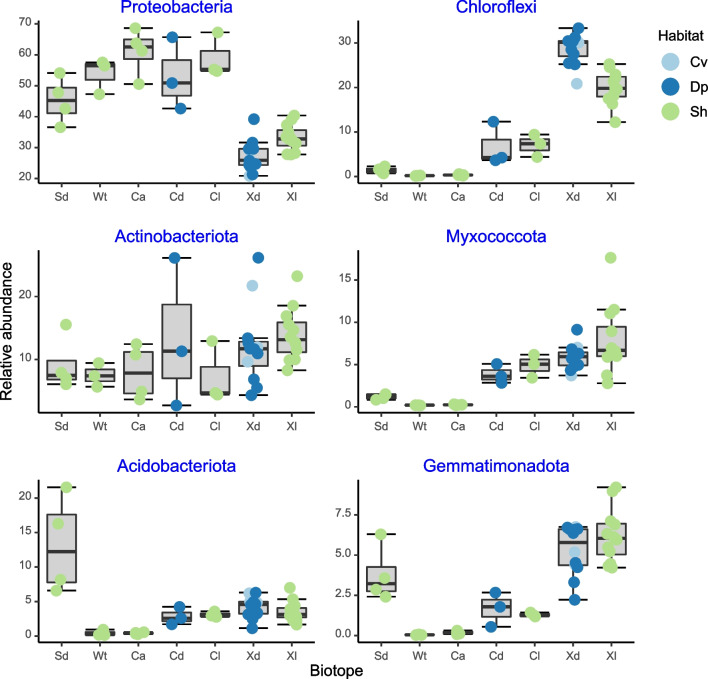
Boxplots showing the relative abundances for the most abundant phyla. Results of GLM analyses are shown after the respective phylum. Proteobacteria: *F*_6,34_ = 22.11, *P* < 0.001, Chloroflexi: *F*_6,34_ = 51.05, *P* < 0.001, Actinobacteriota: *F*_6,34_ = 1.42, *P* = 0.235, Myxococcota: *F*_6,34_ = 28.34, *P* < 0.001, Acidobacteriota: *F*_6,34_ = 12.83, *P* < 0.001, and Gemmatimonadota: *F*_6,34_ = 40.41, *P* < 0.001. The x-axis labels refer to sediment (Sd), water (Wt), *Cinachyrella alloclada* (Ca), *Cinachyrella kuekenthali* from dimly lit (Cd) and illuminated (Cl) habitats, and *Xestospongia muta* from dimly lit (Xd) and illuminated (Xl) habitats. Colored symbols indicate specimens collected from caves (Cv), deep (Dp), and shallow water (Sh)

**Fig. 6:**
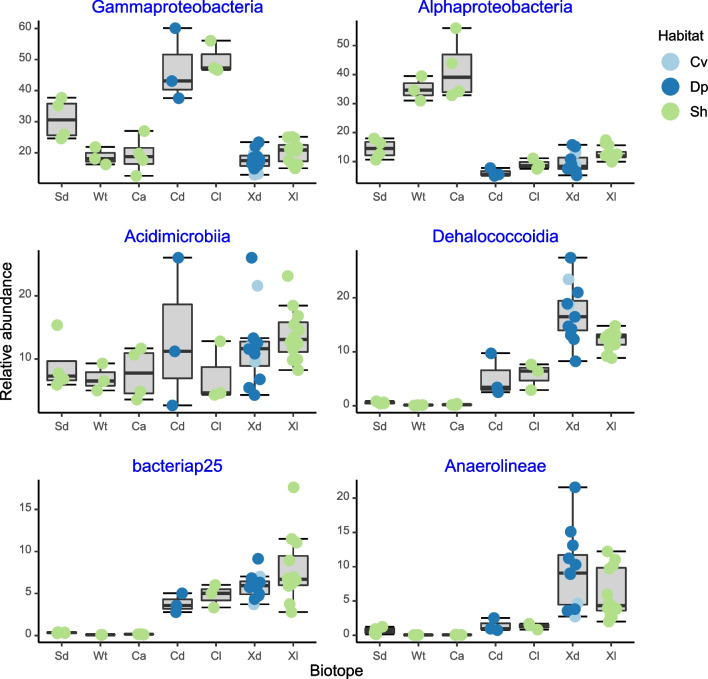
Boxplots showing the relative abundances for the most abundant classes. Results of GLM analyses are shown after the respective class. Gammaproteobacteria: *F*_6,34_ = 25.39, *P* < 0.001, Alphaproteobacteria: *F*_6,34_ = 30.3, *P* < 0.001, Acidimicrobiia: *F*_6,34_ = 1.5, *P* = 0.207, Dehalococcoidia: *F*_6,34_ = 37.53, *P* < 0.001, bacteriap25: *F*_6,34_ = 41.24, *P* < 0.001, and Anaerolineae: *F*_6,34_ = 15.91, *P* < 0.001. The x-axis labels refer to sediment (Sd), water (Wt), *Cinachyrella alloclada* (Ca), *Cinachyrella kuekenthali* from dimly lit (Cd) and illuminated (Cl) habitats, and *Xestospongia muta* from dimly lit (Xd) and illuminated (Xl) habitats. Colored symbols indicate specimens collected from caves (Cv), deep (Dp), and shallow water (Sh)

The eight most abundant phyla, in terms of sequences, together accounted for 86.3% of all sequences and 60.57% of all OTUs (Fig. 4). Boxplots showing variation in the relative abundances of the six most abundant phyla and classes are presented in Figs. 5 and 6, respectively. The relative abundance of Proteobacteria was significantly lower in *X. muta* than in both *Cinachyrella* species (Supplementary data 5). In contrast, the relative abundances of Chloroflexi and Myxococcota were significantly higher in *C. kuekenthali* and *X. muta*, sampled from dimly lit and illuminated habitat, than in all other groups/biotopes. There were no significant differences in the relative abundance of Actinobacteriota among groups. The relative abundances of Acidobacteriota and Gemmatimonadota were significantly higher in sediment, *C. kuekenthali* and *X. muta* than in *C. alloclada* or seawater (Supplementary data 5).

At the class level, the relative abundance of Gammaproteobacteria was significantly higher in *C. kuekenthali* than all other groups and the relative abundance of Alphaproteobacteria was significantly higher in *C. alloclada* and seawater than all other groups (Supplementary data 6). The relative abundances of Dehalococcoidia and bacteriap25 were significantly higher in *C. kuekenthali* and *X. muta* from dimly lit and illuminated habitat than in all other groups. There was no significant difference in the relative abundance of Acidimicrobiia between pairs of groups (Supplementary data 6).

**Fig. 7:**
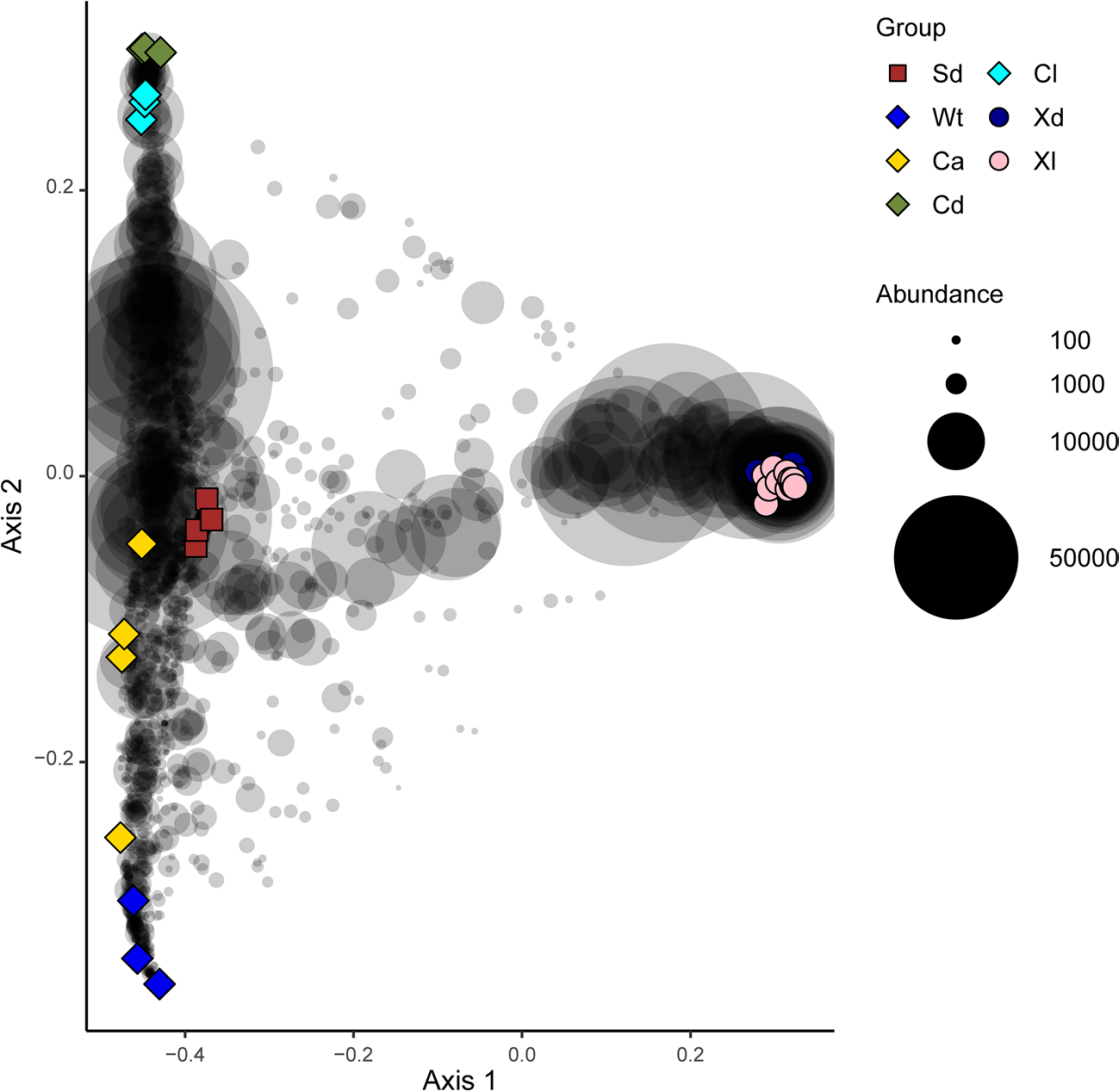
Ordination showing the first two axes of the principal coordinates analysis (PCO) of OTU composition. Symbols are color coded and represent samples from different groups as shown in the legend on the right side of the figure. Gray symbols represent weighted averages scores for OTUs. The symbol sizes for OTUs are proportional to their abundances (number of sequence reads). The eigenvalues for the first and second axes were 5.54 and 0.90, respectively, and explained 48.3 and 7.8%, respectively, of the total variation in the data. The symbols refer to sediment (Sd), water (Wt), *Cinachyrella alloclada* (Ca), *Cinachyrella kuekenthali* from dimly lit (Cd) and illuminated (Cl) habitats, and *Xestospongia muta* from dimly lit (Xd) and illuminated (Xl) habitats

**Fig. 8:**
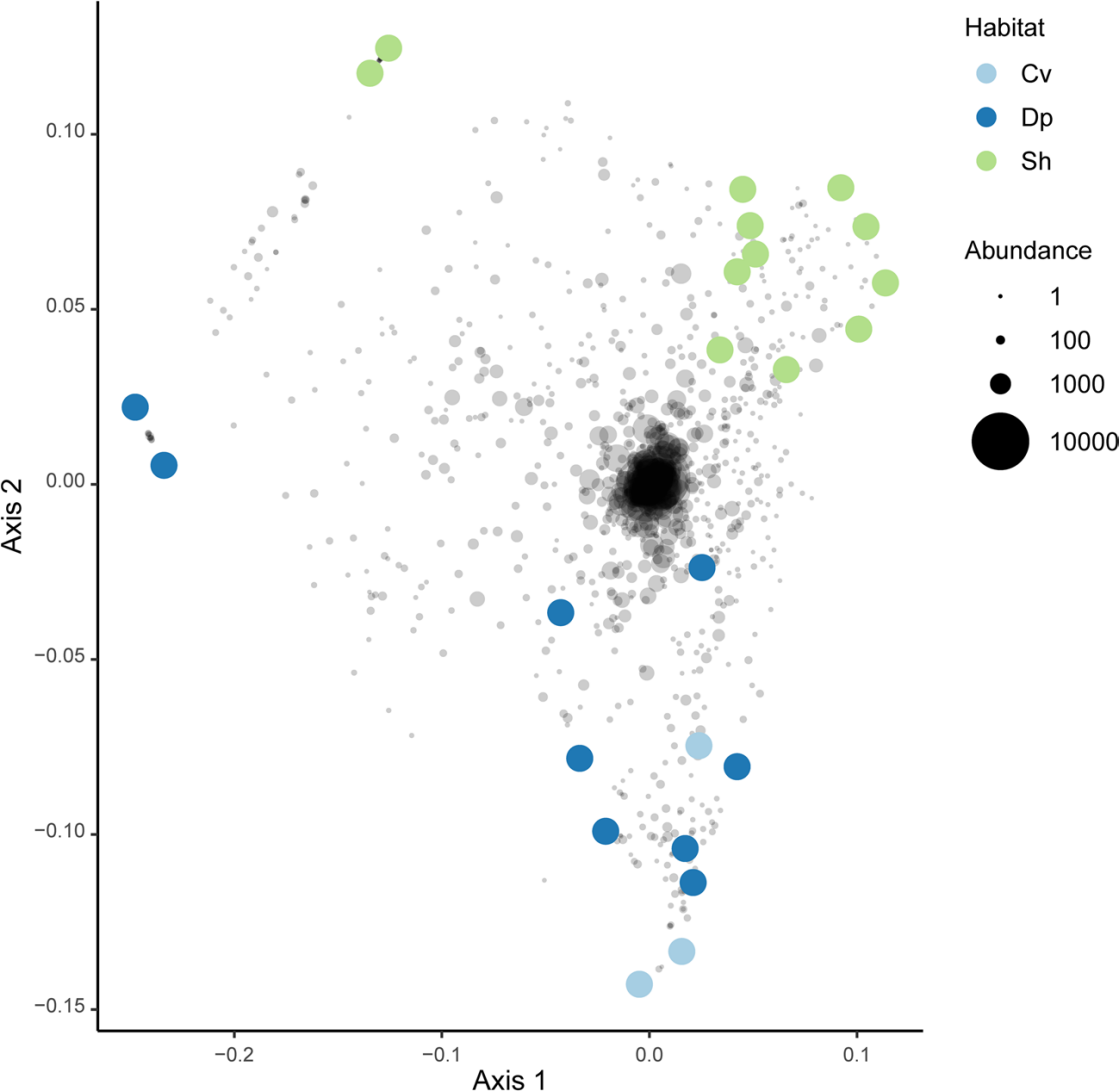
Ordination showing the first two axes of the principal coordinates analysis (PCO) of OTU composition including only samples of *X. muta* collected from caves, deeper, and shallow water. Symbols are color coded and represent samples from different groups as shown in the legend on the right side of the figure. Gray symbols represent weighted averages scores for OTUs. The symbol sizes for OTUs are proportional to their abundances (number of sequence reads). The eigenvalues for the first and second axes were 0.21 and 0.16, respectively, and explained 12.4 and 9.4%, respectively, of the total variation in the data. The symbols refer to *Xestospongia muta* specimens sampled from caves (Cv), deep (Dp), and shallow water (Sh)

There was clear clustering of samples based on biotope (sediment, seawater, and sponge species). The first axis of the ordination separated *X. muta* from the remaining biotopes. The second axis, in turn, separated *C. kuekenthali* from seawater samples with sediment samples and specimens of *C. alloclada* intermediate (Fig. 7). Specimens of *C. kuekenthali* from dimly lit and illuminated habitats also separated along axis 2. The third axis of variation separated sediment from remaining samples while the fourth axis of variation separated *C. alloclada* from *C. kuekenthali* and seawater samples (Supplementary data 7). The group/biotope proved a significant predictor of variation in composition (adonis: *F*_6,34_ = 11.78, *R*^2^ = 0.675, *P* < 0.001). When only including the *X. muta* specimens (Fig. 8), the light habitat (dimly lit versus illuminated) also proved a significant predictor of variation in composition (adonis: *F*_1,22_ = 2.28, *R*^2^ = 0.094, *P* < 0.001).

**Fig. 9:**
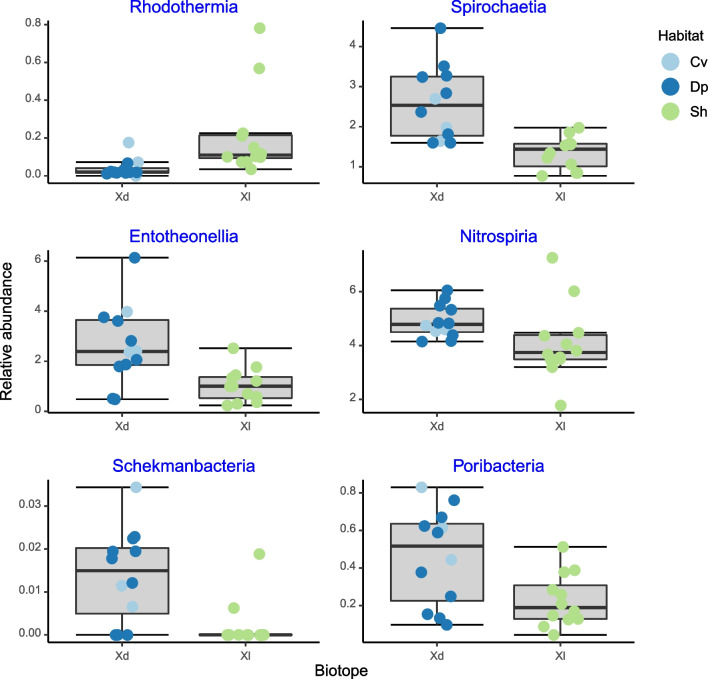
Boxplots showing the relative abundances of bacterial classes, which differentiated between deeper water and cave versus shallow water sponges. The x-axis labels refer to *Xestospongia muta* specimens sampled from dimly lit (Xd) and illuminated (Xl) habitats. Colored symbols indicate specimens collected from cave (Cv), deep (Dp), and shallow water (Sh)

**Fig. 10:**
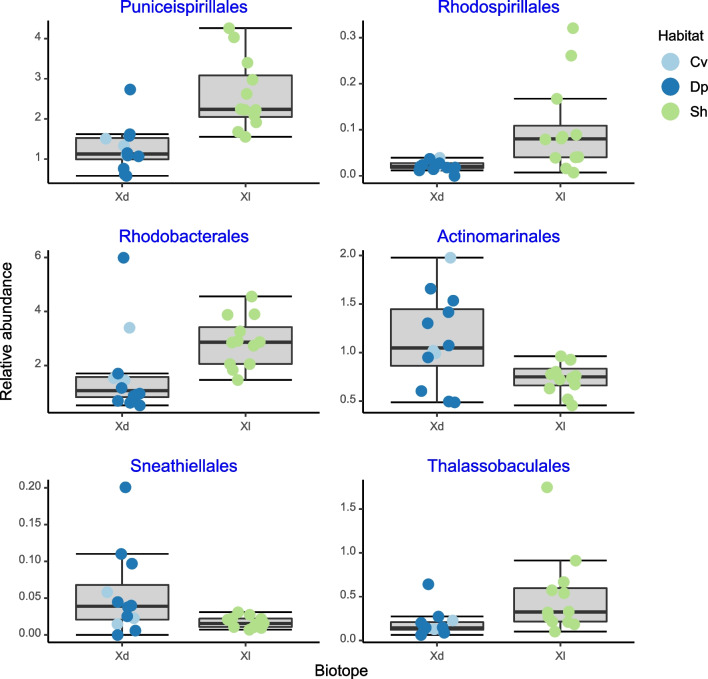
Boxplots showing the relative abundances of bacterial orders, which differentiated between deeper water and cave versus shallow water sponges. The x-axis labels refer to *X. muta* specimens sampled from dimly lit (Xd) and illuminated (Xl) habitats. Colored symbols indicate specimens collected from caves (Cv), deep (Dp), and shallow water (Sh)

Using only *X. muta* specimens, the Boruta analysis yielded nine significant predictive classes and eleven significant predictive orders. Six of the classes and orders with the greatest importance values are presented in Figs. 9 and 10. Other significant predictors included the classes Alphaproteobacteria and Dehalococcoidia (Fig. 6). The classes Alphaproteobacteria and Rhodothermia had greater median relative abundances in shallow water *X. muta* specimens and the remaining classes in specimens sampled from dimly lit habitat. At the order level, all of the alphaproteobacterial orders, with the notable exception of the Sneathiellales, had greater median relative abundances in shallow water specimens. Median Actinomarinales relative abundance was greater in specimens sampled from dimly lit habitat.

**Fig. 11:**
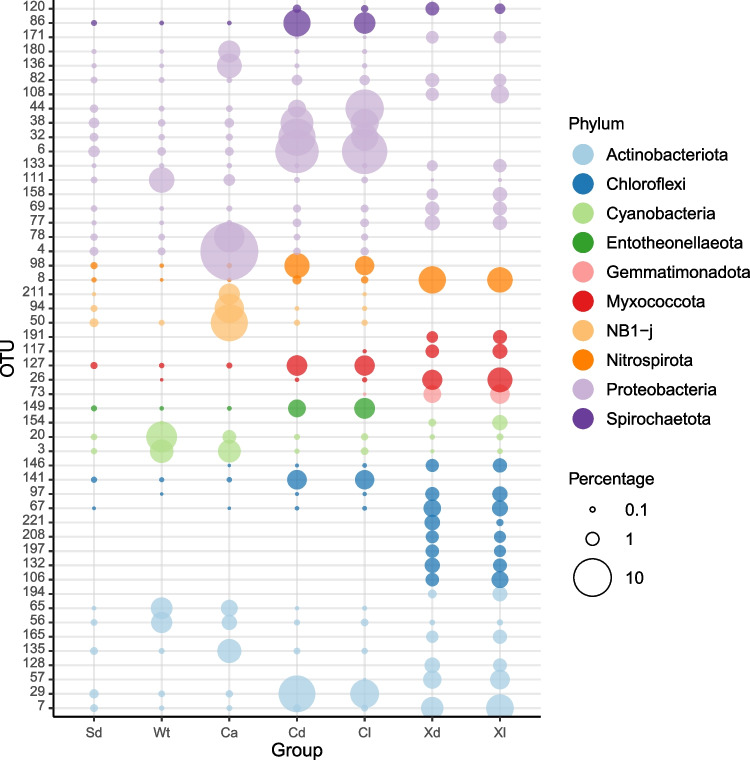
Relative abundance of the most abundant OTUs color coded according to prokaryotic phylum. The x-axis labels refer to sediment (Sd), water (Wt), *Cinachyrella alloclada* (Ca), *Cinachyrella kuekenthali* from dimly lit (Cd) and illuminated (Cl) habitats, and *Xestospongia muta* from dimly lit (Xd) and illuminated (Xl) habitat. The circle size of the OTU is proportional to the mean percentage of sequences per biotope as indicated by the symbol legend at the right of the figure below the phylum assignment legend

**Fig. 12:**
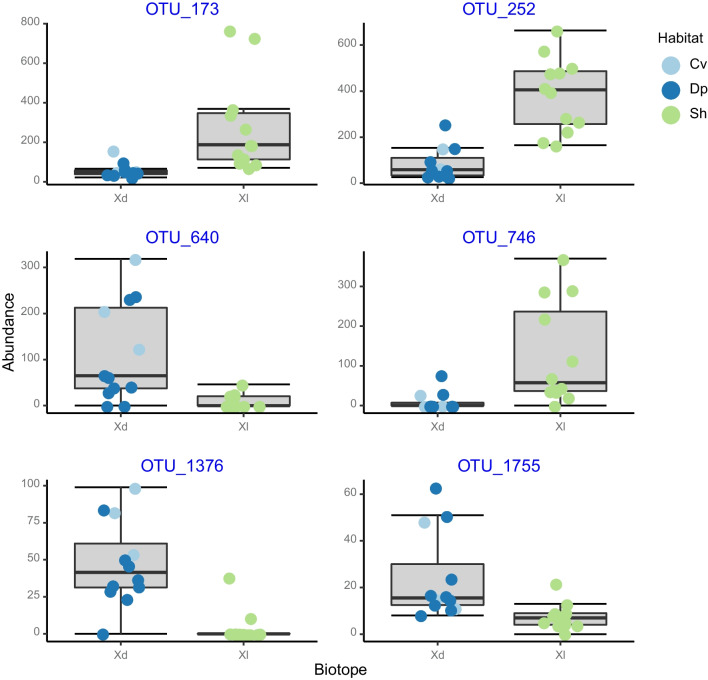
Boxplots showing the relative abundances of OTUs, which differentiated between deeper water and cave versus shallow water sponges. The x-axis labels refer to: *Xestospongia muta* specimens sampled from dimly lit (Xd) and illuminated (Xl) habitats. Colored symbols indicate specimens collected from caves (Cv), deep (Dp) and shallow water (Sh)

There was considerable variation in relative abundance among groups of the 50 most abundant OTUs (Fig. 11). The prokaryotic community of *C. alloclada* differed from seawater and other groups in the higher relative abundances of eight OTUs assigned to the Actinobacteriota (OTU-135), NB1-j (OTUs 50, 94, and 211), Alpha- (OTUs 4, 78) and Gammaproteobacteria (OTUs 136 and 180). *Cinachyrella kuekenthali* housed greater abundances of OTUs assigned to the Actinobacteriota (OTU-29), Chloroflexi (OTU-141), Entotheonellaeota (OTU-149), Myxococcota (OTU-127), Nitrospirota (OTU-98), Spirochaetota (OTU-86), and Gammaproteobacteria (OTUs 6, 32, 38, and 44). OTUs 6, 32, and 44 were all assigned to the *AqS1* genus (Nitrosococcales). The OTUs of *X. muta* were more evenly distributed between dimly lit and illuminated (shallow water) habitats and differed most with respect to the other groups by the abundance of a set of OTUs assigned to the Chloroflexi and three OTUs assigned to the Myxococcota (26, 117, and 191). These OTUs were absent or recorded in very low abundances in the other groups/biotopes. OTUs 106, 132, 197, 208, and 221 were all assigned to the Caldilineales order (Anaerolineae), whereas OTUs 67, 97, and 146 were assigned to the SAR202 clade (Dehalococcoidia). Twenty-two OTUs proved significant predictors of specimens of *X. muta* sampled in dimly lit versus illuminated habitat. OTUs 173, 252, and 746 assigned to the Rhodobacterial genera *Albidovulum*, and *Silicimonas* and the gammaproteobacterial family Woeseiaceae, respectively, were more abundant in shallow water specimens (Fig. 12). In contrast, the OTUs 640, 1376, and 1755, assigned to the Actinomarinales order, *AqS1* genus (Nitrosococcales), and SAR202 clade, respectively, were more abundant in specimens from dimly lit habitat (Fig. 12). Only three OTUs assigned to the Cyanobacteria were included in the 50 most abundant OTUs. OTUs 3 and 20 were assigned to the genus *Synechococcus* and *Prochlorococcus*, respectively. Both of these were most abundant in seawater and *C. alloclada* and were also more abundant in shallow water specimens of *C. kuekenthali* and *X. muta*. OTU-154 was assigned to *Synechococcus spongiarum* and reached greatest abundance in shallow water samples of *X. muta*, where it accounted for 1.22% of the total community compared to 0.14% in deep water specimens and 0.38% in cave specimens. It was not recorded in *C. kuekenthali.*

## Discussion

In the present study, we showed that the prokaryotic communities of three Caribbean sponge species were compositionally distinct from one another and from sediment and seawater. This result is in line with a large number of previous studies highlighting the importance of host identity in structuring prokaryotic community composition [44–48]. The sponges *C. kuekenthali* and *X. muta* housed greater relative abundances of Chloroflexi, Myxococcota, and lower abundances of Alphaproteobacteria than sediment, seawater, and the sponge *C. alloclada*. *Xestospongia muta* was, furthermore, particularly enriched with Chloroflexi members in both the Dehalococcoidia and Anaerolineae classes while Gammaproteobacteria were particularly abundant in *C. kuekenthali*.

In addition to the above, we also showed that specimens of both *C. kuekenthali* and *X. muta* housed compositionally distinct prokaryotic communities in different light habitats. Chloroflexi, for example, were more abundant in specimens of *X. muta* sampled from dimly lit than illuminated habitats, while the reverse held for Alphaproteobacteria. The PCO analysis including only *X. muta* specimens showed that specimens sampled from caves and deeper water largely overlapped, but both were distinct from specimens sampled from shallow water habitat. This suggests that the importance of depth in structuring prokaryotic communities may primarily be related to light attenuation, as opposed to other factors, which vary with depth, such as temperature, at least over the depth range sampled in the present study.

Elsewhere in the Caribbean (Lee Stocking Island, Bahamas, and Little Cayman, Cayman Islands), Morrow et al. [13] studied populations of *X. muta* in shallow water and mesophotic reefs. Using stable isotopes, Morrow et al. [13] showed a change in stable isotope enrichment with depth indicative of a shift from photoautotrophy to heterotrophy. They, furthermore, identified reductions in the relative abundances of Alphaproteobacteria (Rhodobacteraceae and Rhodospirillaceae), Synechococcaceae, and Rhodothermaceae with depth and increased abundances of Entotheonellaceae, Chloroflexi, Poribacteria, and Ectothiorhodospiraceae (Gammaproteobacteria; Chromatiales), although this differed between sites. At one site, SAR202 and Anaerolineae members were abundant members of the microbiome across the whole depth range with the exception of the most shallow samples. At the other site, in contrast, OTUs assigned to the genus *Synechococcus* were abundant with the exception of the deepest specimens where Chloroflexi members dominated thereby highlighting the potential importance of local factors in depth-related shifts in composition across sites. *Synechococcus* members have generally high nitrogen requirements. Morrow et al. [13] attributed the relative stability in Cyanobacteria with depth to increasing NO_x_ concentrations at the site in question such that the increased nitrogen availability compensated for reduced irradiance. They suggested that Chloroflexi may be responding to different environmental parameters at the different sites including photoautotrophy and competition for inorganic carbon with *Synechococcus* members at one site and a shift to other, still-to-be-identified, nutrient sources at the other site, thus explaining the different responses to depth between sites.

Although Morrow et al. [13] observed shifts in composition with depth, the core members of the bacterial communities of *X. muta* remained relatively stable between 10 to 91 m depth. These core members were assigned to the phyla Chloroflexi, Cyanobacteria (*Synechococcus*), Proteobacteria, Actinobacteria, Acidobacteria, Poribacteria, and Thaumarchaeota (*Nitrosopumilus*), and are believed to play key roles in their sponge hosts with respect to host defense and fulfilling nutrient requirements [13]. *Synechococcus* members (including *S. spongiarum*), for example, have been shown to transfer carbon to their host sponges [49].

The results of our study largely confirm those of Morrow et al. [13] in terms of the reduced relative abundance of Alphaproteobacteria with depth and enrichment with other groups such as the Dehalococcoidia, Entotheonellaeota, and Poribacteria suggesting a shift from phototrophy to other functions, e.g., use of dissolved organic matter. In contrast to Morrow et al. [13], the genus *Synechococcus* was only a minor component of the prokaryotic community of *X. muta* in our study, but was more abundant in illuminated than dimly lit habitat.

In order to delve deeper into our data and identify prokaryotic taxa (classes, orders, and OTUs) associated with dimly lit versus illuminated habitat in the sponge *X. muta*, we used an exploratory technique (Boruta) based on the random forest algorithm. The Boruta algorithm can play an important role in studies using next-generation sequencing given the large amount of data involved and a tendency to focus on the most abundant components of the microbial communities. In the present study, use of the Boruta algorithm enabled us to identify a number of predictors at differing levels of taxonomic resolution, some of which may otherwise have been ignored. It is important to remember, however, that the present study is only observational in nature and future studies will be required to explore the mechanisms involved in the host-prokaryotic associations presented here.

The median relative abundances of the classes Alphaproteobacteria and Rhodothermia and orders Puniceispirillales, Rhodospirillales, Rhodobacterales, and Thalassobaculales were greater in specimens from illuminated than dimly lit habitat; the reverse held for the classes Dehalococcoidia, Spirochaetia, Entotheonellia, Nitrospiria, Schekmanbacteria, Poribacteria, and orders Sneathiellales and Actinomarinales.

Rhodothermia, assigned to the Bacteroidota, were mainly represented by the class Rhodothermales and family Rhodothermaceae in the present study. Rhodothermaceae members have been previously obtained from seawater and were reported to carry rhodopsin genes [50]. Another strain obtained from seawater was reported to be gram-negative, obligately aerobic, heterotrophic, and catalase-positive and capable of nitrate reduction [51].

The orders Rhodobacterales, Puniceispirillales, and Rhodospirillales are all members of the Alphaproteobacteria class, a widespread, dominant, and diverse group of free-living and host-associated bacteria [48, 52–54]. The order Rhodobacterales includes members capable of photosynthesis under anoxic conditions [55, 56] and they have been observed in high abundances in corals [45, 57, 58] and sponges [53, 59]. In the present study, Rhodospirillales members were more abundant in seawater than in *X. muta*, indicating that it is possible that they may be part of the transient prokaryotic community of the sponge, rather than the symbiotic host microbiome. Rhodospirillales are commonly found in bacterioplankton communities of coral reefs [60]. They are phototrophic microorganisms [61], which fits with their greater abundance in shallow water specimens. In the present study, the most abundant Puniceispirillales OTUs were either assigned to the SAR116 clade or to the genus *Constrictibacter* (Supplementary Data 3). Members of the SAR116 clade have been previously reported from oligotrophic seawater [62], whereas members of *Constrictibacter* were more abundant in sponges [62]. The prevalence of the SAR116 clade in the bacterioplankton community of seawater [63] suggests that its presence in the sponge microbiome may also be transient. Organisms belonging to SAR116 are normally more abundant in the euphotic zone of the ocean and are known to possess the photoprotein proteorhodopsin [64]. Members of the genus *Constrictibacter* have been reported to transmit vertically in multiple species of the tropical sponge genus *Ircinia* [65] in addition to other eukaryotes [66]. The functional role of the genus *Constrictibacter* is still poorly understood due to the lack of known strains [67].

Dehalococcoidia (phylum Chloroflexi) members have been recorded across varying biotopes including terrestrial soils [68], marine subsurface sediments [69], and sponges [70–72]. Dehalococcoidia members comprise obligate organohalide-respiring species, which is an anaerobic bacterial respiratory process that uses halogenated hydrocarbons as terminal electron acceptors during electron transport [73]. Halogenated hydrocarbons have been detected in various marine sponges, e.g*.,* *Aplysina aerophoba*, *Agelas wiedenmayeri*, and *Agelas conifera*, where they have been suggested to play roles in chemical defense mechanisms including antibiotics and signaling molecules [70, 72]. In the present study, most OTUs assigned to the Dehalococcoidia were also assigned to the SAR202 clade at order level. Busch et al. [74] identified two core OTU members of deep sea sponges using a 90% sequence similarity threshold of which one belonged to the Chloroflexi-Dehalococcoidia-SAR202 clade-hydrothermal vent metagenome and the other to the Actinobacteriota-Acidimicrobiia-Microtrichales-Microtrichaceae-Sva0996 marine group.

The class Schekmanbacteria (assigned to the phylum Schekmanbacteria) was first detected in the metagenome of sediments in the proximity of freshwater aquifers [75]. However, since then, members have been detected in several metagenomes from various marine environments, including in association with marine sponges [6, 76, 77]. Interestingly, multiple studies have reported an increasing presence of Ca. Schekmanbacteria organisms with depth [6, 76]. Functionally, Schekmanbacteria members are thought to be involved in sulfur cycling, as a number of studies have detected genes related to sulfate oxidation and reduction in the genomes of these organisms [78–80].

Members of the class Spirochaetia are frequently reported in association with tropical marine invertebrates, including corals [45, 46, 81] and sponges [45, 46, 82–85]. In sponges of the genera *Tsitsikamma* and *Cyclacanthia*, a bacterium assigned to the genus *Spirochaeta* (class Spirochaetia) was a key member of their core microbial communities and was suggested to have co-evolved with its respective hosts [86]. Others have likewise found a strong cophylogenetic signal between members of the class Spirochaetia and various coral and sponge hosts [81], which suggests that they are symbionts as opposed to transient organisms. Although implied in the production of secondary metabolites [86], little is known regarding the ecological role of Spirochaetia in sponge hosts. In non-marine hosts such as termites, members of the genus *Treponema* (class Spirochaetia) were suggested to function as nitrogen fixers [87], while in the marine environment, *Spirochaeta* appears to metabolize carbon sources in the gills of lucinid clams [88].

In the present study, levels of Entotheonellaeota were similar for *X. muta* and *C. kuekenthali*, but the phylum was practically absent from sediment, water, and *C. alloclada.* In *X. muta*, the median relative abundance was greater in dimly lit than illuminated habitat. Recently, Ruiz et al. [89] found that the bacteria *Candidatus Entotheonella*, a member of this phylum, was among the dominant morphotypes observed in the mesohyl and larvae of the cave dwelling sponge *Plakina kanaky* [89]. The authors suggested that members of this order might be vertically transmitted in *P. kanaky* and, due to their ability to produce unique bioactive compounds, confer an adaptive advantage to the sponge [89]. In fact, there is strong evidence that the genus is responsible for the production of biologically active compounds in the sponge *Theonella swinhoei*, which is known to be a prolific producer of unique bioactive natural products [90]. Together with the work of Ruiz et al. [89], our results hint that members of the Entotheonellaeota phylum may have a relevant ecological role for sponges inhabiting dimly lit habitat.

Poribacteria members were initially associated with a set of sponge species, mainly belonging to the order Verongida [91]. More recently, however, members of the phylum have been found in several other sponge species and other hosts such as corals [92–97]. Among sponges, Poribacteria have mainly been associated with HMA sponges [29, 98]. In the present study, poribacterial abundance was higher in samples of C. *kuekenthali* sampled at greater depths, and was very low in sediment, seawater, and C. *alloclada* (Supplementary data 8).

 Members of the alphaproteobacterial order Sneathiellales deviated from the main trend in the class by having a greater median relative abundance in sponge specimens from dimly-lit habitat. Sneathiellales members have been previously detected in tidal mudflats [99], marine sediment and water [100*–*102], as well as in association with nudibranch and coral hosts [103*,* 104]. The nudibranch-associated Sneathiellales were halotolerant, aerobic, and chemoheterotrophic [103]. Sneathiellales members of Mediterranean seawater communities were shown to be involved in the degradation of polycyclic aromatic hydrocarbons [105]. It is unknown what role they may play in their sponge hosts*.*

Previous studies have observed greater abundances of selected taxa in shallow, euphotic waters. These included Flavobacterales, Rhodobacterales, Actinomarinales, Verrucomicrobiales, Cellvibrionales, and SAR86. In contrast, SAR11, SAR324, SAR202, UBA10353 marine group, Marinimicrobia, Thiomicrospirales, Nitrospinales, and Nitrosopumilaceae were observed to increase in relative abundance with depth [15, 102, 106]. Our results with respect to depth-related differences in members of *X. muta*, appear to, at least partly, overlap observations of depth-related differences in bacterioplankton communities. Note that in the present study, water samples were only sampled from shallow water habitat. In future studies, it would be interesting to sample water from dimly lit habitat in order to assess if potentially transient sponge microbiome members shift in line with the local bacterioplankton community.

## Conclusion

Here, our results showed that the sponge species *C. kuekenthali* and *X. muta* hosted compositionally distinct prokaryotic communities in different light environments. With respect to *X. muta*, our results are in line with a previous study on the impact of depth on bacterial communities of *X. muta* in the Bahamas [13]. Interestingly, our dataset showed that the median relative abundance of the order Entotheonellales was greatest in *Xestospongia muta* specimens collected from dimly lit habitat. This observation and the recent study by Ruiz et al. [89] suggest that members of the Entotheonellales order may play an important ecological role for sponges inhabiting these environments. Members of this order are known for their ability to produce unique bioactive compounds with potential for drug discovery. Future work should clarify this apparent pattern and test if caves, for example, are a hotspot of sponge-associated Entotheonellales with biotechnological potential.

### Supplementary Information

Supplementary data 1. List of samples used in the present study including the sample-id, sample code (Code), group code (Group), Group_full, Species, sampling date (Date), sampling site (Site), Country, Depth (Dep), Latitude (Lat), Longitude (Lon), Country, and Naturalis museum registration number (RMNH POR). (XLS 19 kb)

Supplementary data 2. OTU counts table including taxonomic assignment. (XLS 5426 kb)

Supplementary data 3. List of the 50 most abundant OTUs recorded in the present report and their taxonomic assignment using the Silva (https://www.arb-silva.de/) database. (XLS 12 kb)

Supplementary data 4. Results of emmeans analysis showing pairwise comparisons of differences in selected diversity indices between pairs of groups. (XLSX 11 kb)

Supplementary data 5. Results of emmeans analysis showing pairwise comparisons of differences in the relative abundances of the six most abundant phyla between pairs of groups. (XLSX 15 kb)

Supplementary data 6. Results of emmeans analysis showing pairwise comparisons of differences in the relative abundances of the six most abundant orders between pairs of groups. (XLSX 17 kb)

Supplementary data 7. Ordination showing the third and fourth axes of the principal coordinates analysis (PCO) of OTU composition. Symbols are color coded and represent samples from different groups as shown in the legend on the right side of the figure. Gray symbols represent weighted averages scores for OTUs. The eigenvalues for the third and fourth axes were 0.73 and 0.38, respectively, and explained 6.3 and 3.3%, respectively, of the total variation in the data. The symbol sizes for OTUs are proportional to their abundances (number of sequence reads). The symbols refer to: Sediment (Sd), Water (Wt), *Cinachyrella alloclada*, (Ca), *Cinachyrella kuekenthali* in dimly lit (Cd) and illuminated (Cl) habitats, and *Xestospongia muta* sampled in dimly lit (Xd) and illuminated (Xl) habitats. (PDF 345 kb)

Supplementary data 8. Boxplots showing the relative abundances of HMA indicator taxa. The x-axis labels refer to: Sediment (Sd), Water (Wt), *Cinachyrella alloclada*, (Ca), *Cinachyrella kuekenthali* in dimly lit (Cd) and illuminated (Cl) habitats, and *Xestospongia muta* sampled in dimly lit (Xd) and illuminated (Xl) habitats. Colored symbols indicate specimens collected from caves (Cv), deep (Dp), and shallow water (Sh). (PDF 10 kb)

## Data Availability

Information about the availability of the data reported in this work has been included in the “Material and Methods” section.
