# Supplementary figures and images for: A Study of Sponge Symbionts from Different Light Habitats
Source: Microb Ecol. 2023 Aug 19;86(4):2819–37. doi: 10.1007/s00248-023-02267-x (PMC10640470; doi:10.1007/s00248-023-02267-x)

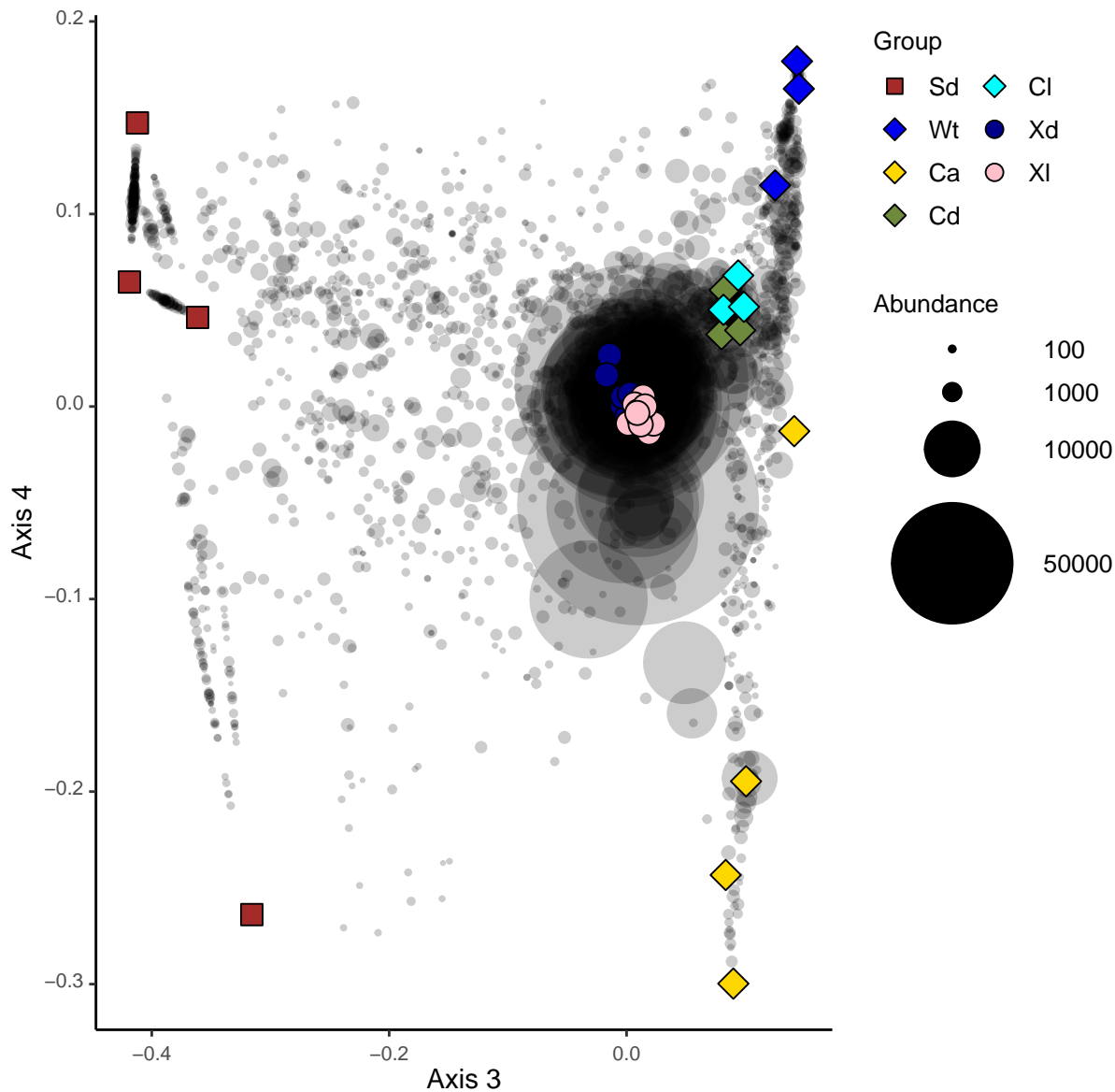

Supplement: Supplementary file 7 — Ordination showing the third and fourth axes of the principal coordinates analysis (PCO) of OTU composition. Symbols are color coded and represent samples from different groups as shown in the legend on the right side of the figure. Gray symbols represent weighted averages scores for OTUs. The eigenvalues for the third and fourth axes were 0.73 and 0.38, respectively, and explained 6.3 and 3.3%, respectively, of the total variation in the data. The symbol sizes for OTUs are proportional to their abundances (number of sequence reads). The symbols refer to: Sediment (Sd), Water (Wt), Cinachyrella alloclada, (Ca), Cinachyrella kuekenthali in dimly lit (Cd) and illuminated (Cl) habitats, and Xestospongia muta sampled in dimly lit (Xd) and illuminated (Xl) habitats. (PDF 345 kb) [file 248_2023_2267_MOESM7_ESM.pdf]
