# Supplementary material for: A Study of Sponge Symbionts from Different Light Habitats
Source: Microb Ecol. 2023 Aug 19;86(4):2819–37. doi: 10.1007/s00248-023-02267-x (PMC10640470; doi:10.1007/s00248-023-02267-x)
Supplement: Supplementary file 8 — Boxplots showing the relative abundances of HMA indicator taxa. The x-axis labels refer to: Sediment (Sd), Water (Wt), Cinachyrella alloclada, (Ca), Cinachyrella kuekenthali in dimly lit (Cd) and illuminated (Cl) habitats, and Xestospongia muta sampled in dimly lit (Xd) and illuminated (Xl) habitats. Colored symbols indicate specimens collected from caves (Cv), deep (Dp), and shallow water (Sh). (PDF 10 kb) [file 248_2023_2267_MOESM8_ESM.pdf]

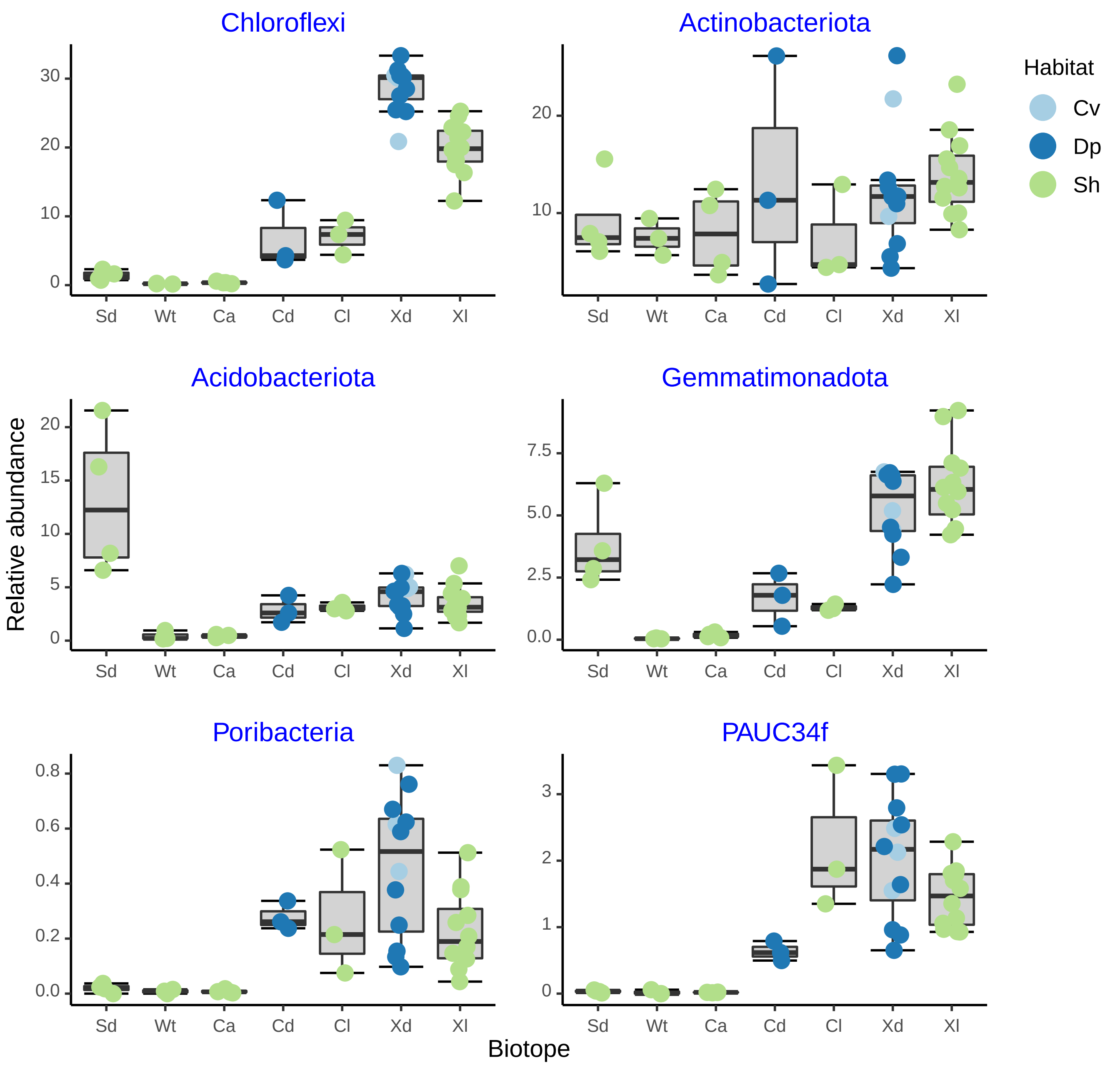

Chloroflexi
Actinobacteriota
Acidobacteriota
Gemmatimonadota
Poribacteria
PAUC34f
Habitat
Cv
Dp
Sh
Sd
Wt
Ca
Cd
Cl
Xd
Xl
Relative abundance
Biotope
